# Targeting vascular leakage in lung inflammation

**DOI:** 10.18632/oncotarget.4907

**Published:** 2015-07-20

**Authors:** Liang Li, Vincent T.K. Chow, Nguan Soon Tan

**Affiliations:** School of Biological Sciences, College of Science, Nanyang Technological University; and Institute of Molecular and Cell Biology, A*STAR; and KK Research Centre, KK Women's and Children's Hospital, Singapore, Singapore

**Keywords:** vascular leakage, lung inflammation

The 2009 outbreak of H1N1 caught many off guard-not least those responsible for tracking the emergence of new influenza strains and outbreaks [1]. Although the influenza A H1N1 pandemic in 2009 proved relatively mild, it still claimed over 14,000 lives across the globe. Given the relentless and rapid mutation of influenza viruses, predicting the onset of pandemics is virtually impossible. Moreover, the development of effective vaccines and antiviral agents against this renegade RNA virus also lags behind the emergence of new influenza strains. Hence, there is considerable interest in strategies that target host responses to combat influenza infection and pneumonia to counter the occurrence of antiviral-resistant strains [1].

Acute lung infection and inflammation such as in influenza pneumonia are associated with the “cytokine storm” that recruits excessive immune cells to infiltrate lung tissue. The infiltrated immune cells clear pathogens by various means such as generating reactive oxygen species that exacerbates tissue damage. Influenza infection also impairs pulmonary functions partially by inducing vascular leakage that facilitates further infiltration of immune cells and pulmonary edema. These phenomena raise the possibility that enhancing lung endothelial barrier integrity during infection and inflammation may ameliorate lung tissue damage, and may be therapeutically useful for acute lung injury [2–4].

Recent studies reveal that angiopoietin-like 4 (ANGPTL4) is upregulated during influenza pneumonia [2]. High-throughput RNA sequencing of lung tissue samples of patients during the 1918 and 2009 influenza pandemics show that ANGPTL4 is one of the most significantly upregulated mRNAs [5]. Our analysis of influenza-infected murine lungs consistently detected elevated ANGPTL4 expression [2]. The expression of ANGPTL4 is also increased in the mouse model of lipopolysaccharide-induced acute lung injury [3]. These observations underscore the importance and conserved role of ANGPTL4 in host responses to infection-induced lung injury. ANGPTL4 is a host protein belonging to the angiopoietin-like protein family, and is implicated in context-dependent angiogenesis [6]. Its function is mediated via a Tie2-independent mechanism. ANGPTL4 modulates vascular junction integrity by integrin signaling and disruption of intercellular VE-cadherin and claudin-5 interaction [7]. ANGPTL4-deficient mice or immuno-neutralization of ANGPTL4 reduces lung tissue leakiness and damage in influenza-infected mice [2]. Similarly, ANGPTL4 suppression by siRNA also protects mice from lipopolysaccharide-induced acute lung injury [3]. Interestingly, a recent study adopting a similar strategy to decrease vascular leakage but against a target different from ANGPTL4, also improves survival of mice with severe influenza infection, i.e. via administration of a Tie2-agonist tetrameric peptide (Vasculotide) [4]. Although the above studies employed different models, the common theme of the interventions aims to improve vascular integrity, culminating in better outcomes.

Bacterial pneumonia may present as a primary disease process, or as the final terminal event in individuals who are already debilitated. A historical review of the 1918 influenza pandemic suggests that the majority of deaths were not directly attributed to the influenza virus; rather the mortalities were due to bacterial co-infections. While the objective of anti-inflammatory approaches against influenza pneumonia is to mitigate the devastating impact of hypercytokinemia, such therapies also render the host more susceptible to secondary infections. In contrast, direct regulation on vascular or tissue leakiness may attenuate excessive inflammation without compromising host immune defenses. Although the role of ANGPTL4 in inflammation remains to be fully elucidated, our interrogation of microarray gene expression data of lungs from influenza-infected mice treated with neutralizing ANGPTL4 antibody did not reveal significant diminution of host defenses against bacterial infections. Hence, improving vascular integrity may offer a more effective and probably safer alternative to combat lung infection. Furthermore, ANGPTL4 also shows great potential as a biomarker for pneumonia.

Vascular leakage is a hallmark of many infectious diseases, including those caused by dengue virus, severe acute respiratory syndrome (SARS) coronavirus, and Middle East respiratory syndrome (MERS) coronavirus. The roles of ANGPTL4 in these infections are unclear, and certainly warrant future investigations. Importantly, the recent studies on host response factors such as ANGPTL4 provide a new level of understanding with respect to vascular integrity in pulmonary infection and inflammation.
